# Maternal Perception of a Child with Cancer

**DOI:** 10.34763/jmotherandchild.20212502.d-21-00010

**Published:** 2022-04-01

**Authors:** Ida P. Opalińska

**Affiliations:** 1 Faculty of Psychology, University of Warsaw, Warsaw, Poland

**Keywords:** Representation, childhood cancer, mother-child relationship, cancerous disease

## Abstract

**Background:**

The representation of a child is an important element of mother-child relations, which allows the mother to empathise with and respond to the child’s needs. A child’s cancer may be reflected in the mother’s representation of the child. The aim of this study was to see whether there were differences in a mother’s representation of healthy and oncologically ill children.

**Material and methods:**

The participants in the study include 30 mothers of oncologically ill children and 25 women with healthy children as the control group. The study used a self-constructed questionnaire containing questions about the mothers’ representations of their children as well as demographic information.

**Results:**

Women with oncologically ill children less frequently described their children as independent, impulsive, needing food and the recognition of others, than mothers of healthy children. They also felt fear more frequently when thinking about the child.

There were, however, common elements in representations of ill and healthy children. Impulsiveness and interest in computer games and movies was more often attributed to boys than girls in both categories, while helpfulness was attributed more often to older children than younger ones. Single mothers felt regret more often when thinking about the child than mothers who had some helpers.

**Conclusion:**

Maternal representations of a child may include, but do not have to include, disease-related content. Perceptions of a child’s independence, impulsiveness, and needs seem to be related to the child’s health, but for other elements of the child’s image this relation is not present.

## Introduction

The most common cancers in children are leukemias, lymphomas, and tumours of the central nervous system [1,2]. Childhood cancer is life-threatening and requires significant changes in the daily functioning of the ill child and their family; therefore it can be considered a crisis situation [3].

A child or adolescent who is sick or undergoing hospital treatment requires constant care from the medical staff and parents, which is associated with loss of autonomy for young patients, changes in family life, a loss of contact with peers, and oftentimes with changes in their own sense of identity and integrity [4,5].

## Representations of the child in a mother’s mind

For the purposes of the conducted research, the definition of *representation* as an image of the child consisted of elements such as its features, needs, and preferences – the cognitive aspect of representation, as well as the mother’s emotional relationship to the child – the emotional aspect [6,7,8,9].

The function of the representation of the child created in the mother’s mind is to understand and satisfy the child’s needs, and the creation of the representation influences the regulation of the child’s state of arousal through the mother, which processes and regulates content that is too difficult for the child to cope with by themselves [10,6,8]. Recognising the child’s own ideas, thoughts, expectations, and experiences allows the mother to experience closeness with the child, empathise with the child’s states, and satisfy the child’s needs [11]. A mother who feels that her child is similar to her has the ability to understand her child. However, a mother who feels that her daughter or son is clearly different from her, for example due to an illness, have difficulty experiencing a sense of closeness with their child and understanding their child’s needs [8]. On the other hand, a mother’s perception of her child’s individuality is a factor that eases separation [11].

## Representation of a frequently ill child and a healthy child in a mother’s mind

Despite lengthy research, it was not possible to find results that would directly refer to the parent’s representation of a child with cancer. However, such studies have been carried out for maternal representations of children in the context of chronically ill and obese children. Women from this group create less detailed descriptions of their sons and daughters than mothers of healthy children; their narration more often focuses on the child’s somatic state, and contains less about the child’s cognitive and socioemotional functioning [8,12]. They also usually have an ambivalent or negative emotional attitude towards their children [8,13].

Moreover, mothers of ill children define their sons and daughters as less independent than healthy children, and have difficulty realising the psychological distinctiveness of the child [14,8,7,12]. Some mothers of ill children also have difficulty modifying a child’s image and have a tendency to deny their children’s illness [10].

Mothers of healthy children create descriptions of their daughters and sons referring to a greater number of domains than do women whose child is sick [7,8,12]. Their representation of the child consists of child’s personality traits, abilities, needs, preferences and their own experiences from the relationship with the child [7,8,10, 11,12]. Mothers of healthy children also have rather positive attitudes towards their children [7,8,13].

The aim of the study was to analyse the differences in representations by mothers of childrenwith cancer and mothers of healthy children. The following research questions were formulated:

- Will mothers of healthy children and mothers of oncologically ill children differ in terms of their representations of their children?
- Will there be some specific elements appearing only (or less or more frequently) in the representation of the oncologically ill child and not in the representation of the healthy child?
- Will there be some common elements of the representations of ill and healthy children?

## Material and methods

### Participants

Thirty mothers with children treated for leukemia, tumours and other types of cancer took part in the study from the Department of Oncology and Hematology at the Świętokrzyskie Paediatric Center, named after Władysław Buszkowski, in Kielce, Poland and the Children’s and Adolescents’ Oncology and Surgery Clinic at the Mother and Child Institute in Warsaw, Poland. Women were informed that the survey was anonymous and the results would be presented for groups, not individuals. They were assured that they could quit anytime, even at the end of the survey. They also had the opportunity to leave an email on a separate form in order to be informed about group results of the research.

The control group consisted of 25 mothers of healthy children and was selected in order to match the parameters of the group containing the ill children (age of the mother, the number of children, the age and sex of the ill child, and their birth order). More detailed information about the groups is stated in Table 1.

**Table 1:** Information about the experimental and control groups

	Mothers of oncologically ill children	Mothers of healthy children
Number of mothers	30	25

Mothers’ age	21 to 54 years old (M = 38. 93, SD = 8.71)	23 to 56 years old (M = 38.04, SD = 8.27)

Number of children	1 to 5 children:	1 to 5 children:
	• most mothers had 1 or	• most mothers had 1 or
	2 children	2 children
	• 6 mothers had 3	• 5 mothers had 3
	children	children
	• 3 mothers had 4 children	• 1 mother had 4 children
	• 2 mothers had 5 children	• 2 mothers had 5 children

Age of the children	2 to 18 years old (M = 10.70, SD = 5.02)	2 to 18 years old (M = 9.82, SD = 4.93)

Sex of the children	16 boys and 14 girls	15 boys and 10 girls

Birth order of the child	• most children were the oldest or youngest in the family	• most children were the oldest or youngest in the family
	• 8 were the only child	• 8 were the only child
	• 1 was the middle child	• 1 was the middle child

Was the mother the only	• Yes - 16 mothers	• Yes - 5 mothers
person to take care of the child?	• No - 14 mothers	• No - 20 mothers

Other caregivers of the child, who helped the mothers	• fathers and grandparents (most of the mothers)	• fathers and grandparents (most of the mothers)
	• other family members	• other family members
	• friend of the mother (1 person)	• friend who had a child of similar age (1 person)
	• daughter's boyfriend (1 person)	

Time since diagnosis	2 months to 9 years	-

Chronic disease experience in the family	• the child or another person in the family had already suffered from a chronic disease (17 mothers)	-
	• the child's cancer was the first such situation in the family (12 mothers)	
	• no answer (1 mother)	

## Measures and procedure

A questionnaire (presented in the Appendix) was created for the study to examine the representation of a child in the mother’s mind, containing questions about the child’s personality, needs, preferences and emotional relationship of the mother, in which the respondents indicated all items on an answer list, accurately defining their children and their own feelings. The response lists were created on the basis of the Polish Adjective List [15], Abraham Maslow's theory [16], the Multidimensional Preferences Questionnaire [17] and the Scale of Emotional Intelligence - Faces [18]. The accuracy of the items on the lists was assessed by 12 competent judges (psychologists). Only those items which at least 9 out of 12 judges (75%) assessed as corresponding to the constructs of personality, needs, preferences and emotional relationship, qualified for the final version of the questionnaire.

In addition, the mothers participating in the study completed a demographic questionnaire (presented in the Appendix).

## Statistics

The relationship between the controlled variables (mother's age, number of children, age of the described child, child's sex, the child's birth order, time since diagnosis, experience of a chronic disease in the family, who cares for the child) with the mother's representation of the child was checked. For this purpose, chi square tests were performed and Kramer's V correlations were calculated for variables on nominal scales (child's sex, birth order, experience of a chronic disease in the family, who cares for the child, representation of the child including the child's personality traits, emotions felt at the thought of the child, interests and needs of the son or daughter). Spearman rho correlations were calculated for quantitative variables that did not have a normal distribution (mother’s age, child's age). The Bonferroni correction was also used in order to eliminate random significant correlations.

In order to find out whether there are differences in the representations of mothers with healthy and sick children, chi square tests with Bonferroni's correction were performed for nominal variables describing personality, needs and interests of children, and mothers' emotions when thinking about children.

## Results

The relationships between the child's sex, birth order, and whether the mother is the only person caring for the child were shown to be related to some elements of the mother's representation of the child. The results are shown in Table 2.

**Table 2:** Relationship between demographic traits and elements of mother’s representation of the child

Element of the representation	Result
Impulsiveness	more often boys than girls (V = 0.38, p < 0.01)
Interest in computer games	more often boys than girls (V = 0.47, p <0.01)
Interest in movies	more often boys than girls (V = 0.28, p <0.05)
Helpfulness	more often the oldest children than youngest or middle (V = 0.50, p <0.01)
Regret	more often single mothers than women who had someone helping with their care (V = 0.55, p <0.05)

## Differences between mothers of healthy and sick children in terms of representation

Differences were demonstrated in the representation of children by mothers from the experimental and control groups in terms of their personality and needs, as well as the mother’s emotions related to the child. Results are shown in Table 3.

**Table 3:** Differences between the representation of the oncologically ill child and the healthy child

Element of the representation	Frequency in representation of ill child compared to healthy child
Impulsiveness	less often (chi square = 5.40, p <0.05)
Independence	less often (chi square = 5.11, p <0.05)
Need of food	less often (chi square = 13.99, p <0.001)
Need of recognition of others	less often (chi square = 9.63, p <0.01)
Fear	more often (chi square = 6.42, p <0.05)

## Discussion

The current study revealed that mothers of children with cancer are less likely to describe their daughters and sons as independent, and more often declare fear at the thought of them, than mothers of healthy children. These results are consistent with data from previous research about the representation of children that mothers of frequently ill children have [8,19,13,12]. Mothers whose children were frequently ill described their children as less independent than healthy children and more often had an emotionally ambivalent attitude towards their children than mothers whose sons or daughters did not experience frequent illnesses.

In the representation regarding the needs of a child with cancer, mothers less frequently indicated food compared to the representation that mothers with healthy children had. This result corresponds to the picture of ailments experienced by children with cancer during some phases of the treatment. Among the more severe effects of treatment, children mention nausea and vomiting, which make it difficult to eat [20].

The present study also showed that mothers of children with cancer less frequently described their sons and daughters as impulsive and less frequently mentioned recognition by others among their needs, as compared to mothers of healthy children, which has not appeared in previous studies. Earlier analyses of the maternal representation of chronically ill children mainly focused on comparing the level of described detail in the narrative of mothers of ill and healthy children, revealing their emotional relationship to the child and the categories around which the descriptions were focused, and less on the individual traits and properties that mothers attributed to their children [8,9,13,12], hence subtle differences in the frequency of assigning particular traits or interests to children could have been obscured. In addition, the current study is one of the first on this topic to use a list of words from which mothers chose terms that suited their children and the emotion they felt when thinking about their children, rather than analysing the open-ended statements of mothers. The change in the form of research could have influenced the appearance of terms that mothers did not mention in open-ended statements, or did very rarely; such terms became perhaps more cognitively accessible to them thanks to a ready-made list of terms in this study.

The results of the study show that the maternal representation of ill children does not differ significantly in most aspects from the representation of healthy children. There are common features for both groups. Women more frequently attribute impulsiveness and interest in computer games and movies to their sons than their daughters, and they attribute helpfulness as a personality trait to their older children more often than to the youngest or middle child, which may be related to the development of pro-social behavior as children grow older. Mothers who are the only person caring for a child more frequently felt regret when thinking about the child than mothers who had help in care, which may be related to single mothers focusing more on their children, but also their stress and fatigue caused by being the only one who takes care of the child.

## Study limitations

Only 30 mothers of children with cancer and a control group of 25 mothers of healthy children participated in the study. Moreover, the experimental group consisted of mothers whose children were treated in only two hospitals in Poland, in which children suffering from leukemia are most often treated, which has the best prognosis of all childhood cancers. Thus, the examined mothers do not fully exemplify the group of mothers of children with cancer. The situation of being in hospital may be traumatising for the mother and child due to its unpredictability, which may affect the functioning of the mother, including the cognitive availability of her own experiences, and thus the results of the study might not be generalisable, and future research is needed.

## Conclusions

The obtained results show that the representation of a child with cancer may, but does not have to, include disease-related content.

Mothers of children with cancer less frequently perceived independence and needs related to food and recognition by others than mothers of healthy children, which may affect the child’s development (seeing a child’s independence has an impact on the separation process and picturing a child’s needs affects the way the mother responds to them). The question arises: how much is this representation accurate to the actual child’s experiences? Psychologists and other health specialists could examine this topic with the mothers.

However, there are also similar elements in the content of the representation of ill children and healthy children. Further researches are needed to know what determines that some contents of the child’s image seem to be associated with the dimension of the illness in the child, while the other elements are not.

## Key points

- A child’s cancer is a crisis, a traumatic situation, and can be reflected in the image of the child that the mother has.
- Mothers of oncologically ill children less frequently described their sons and daughters as independent and impulsive than mothers of healthy children.
- Women whose children were treated for cancer mentioned food and recognition by others less frequently among the children's needs.
- Psychologists and other specialists can help mothers to compare their representations with the child’s actual experiences.
- Some elements of the child’s image are common for both mothers of oncologically ill children and mothers of their healthy peers.
- Future research is needed to examine why some elements of the representation of the child seem to be associated with cancer, while others are not, and how this affects the mother-child relationship.

## Appendix

### Questionnaire used in the research

Please look at the list of adjectives below and circle all that you would use to describe your child:

- Canny
- Intemperate
- Sensitive
- Shy
- Creative
- Careless
- Dynamic
- Systematic
- Thoughtful
- Dutiful
- Independent
- Helpless
- Fearful
- Helpful
- Caring
- Hot-tempered
- Frivolous
- Argumentative
- Conscientious
- • Emotional
- Egoistic
- Self-possessed

Please think about your child for a while and then circle all the words that describe your feelings during thinking of the child:

- Satisfaction
- Reluctance
- Amusement
- Annoyance
- Pride
- Concern
- Gratitude
- Awkwardness
- Guiltiness
- Embarrassment
- Joy
- Sadness
- Surprise
- Relief
- Daze
- Anger
- Fear
- Favor
- • Enchantment
- Euphoria
- Adoration
- Anxiety
- Regret
- Excitation
- Enthusiasm
- Enjoyment

From the interests stated below, please circle all the things that your child likes:

- Trains
- Handicrafts
- Watching sport
- Cooking
- Films
- Animals
- Fashion
- Music
- Hairdressing
- Computer games
- History
- Travel
- Visual arts
- Dancing
- • Photography
- Books
- Cars
- Doing sport
- Learning new languages
- Other (what?)……………………………………………………………………………

Please think for a while and then circle from the propositions below all the things that your child needs to feel good:

- Rest
- Safeness
- Being loved
- Food
- Breaking free from stress
- Calm
- Friendship
- Recognition by others
- Spirituality
- Staying healthy
- Having a purpose
- Bond with other people
- Self-esteem
- Being accepted
- Support
- • Being understood

### Questionnaire used in the research

Please write the right numbers in the questions below:

Age: ………..

Number of children:…………..

Age of the described child:…………

Time from the diagnosis 1 This question was only in the questionnaire for the mothers of oncologically ill children. :…………

Please mark the correct answers in the questions below:

Sex of the described child:

□ Boy

□ Girl

If the described child is not your only child, is he/she:

□ The youngest

□ Middle

□ The oldest

Did your child or other person from the family suffer from some chronic disease? 1 This question was only in the questionnaire for the mothers of oncologically ill children.

□ Yes

□ No

Are you the only person taking care of the child?

□ Yes

□ No

If not, who besides you takes care of the child? (Please write down the person/people)

…………………………………………………………………………………………………
